# WELL-BEING AND RESILIENCE TO DEMENTIA-RELATED NEUROPATHOLOGY

**DOI:** 10.1093/geroni/igac059.343

**Published:** 2022-12-20

**Authors:** Emily Willroth, Bryan James, Eileen Graham, Alifiya Kapasi, David Bennett, Daniel Mroczek

**Affiliations:** Washington University in St. Louis, St. Louis, Missouri, United States; Rush University, Chicago, Illinois, United States; Northwestern University, Chicago, Illinois, United States; Rush Alzheimer’s Disease Center, Chicago, Illinois, United States; Rush University, Chicago, Illinois, United States; Northwestern University, Chicago, Illinois, United States

## Abstract

Not all older adults with dementia-related neuropathology in their brains experience cognitive decline or impairment. Instead, some people maintain relatively normal cognitive functioning despite neuropathologic burden, a phenomenon called cognitive resilience. Using a longitudinal, epidemiological, clinical-pathologic cohort study (N=349), the present research investigated associations between well-being and cognitive resilience. Consistent with pre-registered hypotheses, higher eudaimonic well-being (i.e., Ryff Psychological Well-being Scale) and higher hedonic well-being (i.e., Satisfaction with Life Scale) were associated with better-than-expected cognitive functioning and less-than-expected cognitive decline relative to one’s neuropathological burden (i.e., beta-amyloid, neurofibrillary tangles, Lewy bodies, vascular pathologies, hippocampal sclerosis, and TDP-43). The association of eudaimonic well-being in particular was present above and beyond known cognitive resilience factors (i.e., socioeconomic status, education, cognitive activity, low neuroticism, low depression) and dementia risk factors (i.e., ApoE genotype, medical comorbidities). This research highlights the importance of considering eudaimonic well-being in efforts to prevent dementia.

